# Interaction between avian influenza subtype H9N2 and Newcastle disease virus vaccine strain (LaSota) in chickens

**DOI:** 10.1186/s12917-018-1689-4

**Published:** 2018-11-20

**Authors:** Hany F. Ellakany, Ahmed R. Gado, Ahmed R. Elbestawy, Hatem S. Abd El-Hamid, Hafez M. Hafez, Mohamed E. Abd El-Hack, Ayman A. Swelum, Abdullah Al-Owaimer, Islam M. Saadeldin

**Affiliations:** 1 Department of Poultry and Fish Diseases, Faculty of Veterinary Medicine, Damanhour University, Elgomhoria st, 63, Damanhour, Elbehira 22511 Egypt; 2 Institute of Poultry Diseases, Free University Berlin, Königsweg, 63, 14163 Berlin, Germany; 3 Department of Poultry, Faculty of Agriculture, Zagazig University, Zagazig, 44511 Egypt; 4 Department of Animal Production, College of Food and Agriculture Sciences, King Saud University, P.O. Box 2460, Riyadh, 11451 Saudi Arabia; 5 Department of Theriogenology, Faculty of Veterinary Medicine, Zagazig University, Zagazig, 44511 Egypt; 6 Department of Physiology, Faculty of Veterinary Medicine, Zagazig University, Zagazig, 44511 Egypt

**Keywords:** Interaction, Avian influenza, H9N2, LaSota, NDV

## Abstract

**Background:**

H9N2 avian influenza virus is endemic in Egyptian poultry flocks. The role of the live viral vaccines such as LaSota in exaggeration of the clinical picture of H9N2 infection under field conditions is significantly important leading to severe economic losses due to higher mortality and lower growth performance. This experiment was designed to identify the possible interaction between experimental infection with H9N2 virus and NDV live vaccine (LaSota strain) in broiler chickens. Six groups each of 20 broiler chicks were used. Three groups (G1–3) were infected with H9N2 and vaccinated with LaSota, 3 days before, at the same day or 3 days post vaccination (dpv), while the remaining groups (G4–6) were non-vaccinated infected, vaccinated non-infected and non-vaccinated non-infected.

**Results:**

The highest mortality rate (37.5%) was noticed in chickens of G1 (H9N2 infected 3 days prior LaSota vaccination). Also, this bird group had the most severe clinical signs, histopathological lesions and the longest viral shedding for 9 days post infection (dpi). In the 2nd and 3rd groups, the mortality rate was the similar (31.2%) with less pronounced clinical signs, histopathological lesions and H9N2 shedding was for only 6 dpi with the least shedding quantity in chickens of G3. The control non-vaccinated infected chickens (G4) had 18.7% mortality with the least degree of clinical signs, lesions and the highest viral shedding quantity but only for 6 dpi. At 35 days of age, there was a statistical significant decrease (*P* < 0.05) in chicken’s body weight of all H9N2 infected groups from G1 to G4 compared to non-infected control groups, G5 and G6 respectively.

**Conclusion:**

It was clear that laSota vaccination significantly affect H9N2 infection in broiler chickens regarding clinical signs, mortality rate, lesions, performance and viral shedding.

## Background

Low pathogenic avian influenza (LPAI) subtype H9N2 virus is widespread worldwide, being endemic in poultry populations in Asia and the Middle East. The first isolation of H9N2 virus in Egypt was obtained from the apparently healthy commercial bobwhite quail flock (A/quail/Egypt/113413v/2011/H9N2) in May 2011 [17]. Later on, the virus was isolated from commercial broiler, broiler breeder and layer flocks [27, 32]. H9N2 AIV infection in broilers cause reduction of growth rate and adverse the feed conversion ratio. In addition, high mortality rate appeared in case of co-infection with other pathogens such as infectious bronchitis virus (IBV), Newcastle disease virus (NDV), *Staphylococcus aureus*, Ornithobacterium rhinotracheale and *E. coli* or immune suppression, which exacerbates H9N2 influenza A virus infection in chickens [18, 23]. Lentogenic NDV strains are commonly used as live vaccines in the commercial poultry industry to protect from virulent forms of NDV. Experimental infections of specific pathogen-free (SPF) chickens with NDV vaccine strains cause little to no clinical disease. However, the application of the vaccine under field conditions can decrease productivity by inducing mild respiratory symptoms, which could be exacerbated concomitantly with other respiratory pathogens or in combination with environmental stressors [33]. Viral interference is a phenomenon explained previously [13], in which the infected cells by a virus do not permit multiplication of a second virus. Both viruses (LPAI and NDV) replicate in the epithelial cells of the respiratory and intestinal tracts (where trypsin-like enzymes is present) through competing for target cells or replicating in adjacent cells exacerbating clinical signs of disease in infected birds or produce viral interference, masking infections in one of them [9, 38].

In this experiment, we studied the interaction between LPAI subtype H9N2 infection and NDV vaccine (LaSota strain) in broiler chickens regarding clinical signs, body weights, post-mortem lesions, mortality, viral shedding, histopathological lesions and serological response for both viruses.

## Results

### Clinical signs (Table 1)

The observed signs in all infected groups with H9N2 virus (G1–4) were depression, decreased feed consumption and respiratory signs. The most severe clinical signs in terms of morbidity were observed in chickens of G1, while the least were in chickens of G4 as the respiratory signs started by sneezing and coughing then exaggerated as rales, nasal discharge, and head swelling till the end of observation period (21 days). Also, white greenish diarrhea was observed from 3rd dpi and subsiding at 11th -12th dpi. In addition, only in G1 exhibited thick oral mucous, which started at 7th dpi and continued until end of observation period.

**Table 1:** Degree of severity of clinical signs

Chicken groups	G1	G2	G3	G4	G5	G6
Clinical signs						
Sneezing	+++	++	++	+	–	–
Coughing	+++	++	++	+	–	–
Rales	+++	++	++	+	–	+
Head swelling	+++	++	+	–	–	–
Diarrhea	+++	++	++	+	–	–
Depression	+++	++	++	+	–	–
Thick oral saliva	+++	–	–	–	–	–

-: No signs +: Mild degree ++: Moderate degree +++: Severe degree

### Daily mortality (Table 2) and post-mortem (PM) lesions

Chickens of G1 suffered from early highest mortality 6/16 birds (37.5%) from 7th to 17th dpi compared to 31.2% (5/16 birds) in both G2 and G3 which began from 8th to 15th and 2nd to 9th dpi, respectively in both groups. While, in G4, the mortality rate was 18.7% (3/16 birds) with delayed start at day 12th dpi and continued only for 3 days.

**Table 2:** Mortality rates in all experimental chicken groups from 15 to 35 DO after infection with LPAI subtype H9N2 alone or in combination with LaSota vaccine

Age (days)	dpi	Daily mortality/each chicken group					
		1	2	3	4	5	6
15	1	–	–	–	–	–	–
16	2	–	–	–	–	–	–
17	3	–	–	–	–		–
18	4	–	–	–	–	–	–
19	5	–	–	–	–	–	–
20	6	–	–	–	–	–	–
21	7	2	–	–	–	–	–
22	8	1	–	–	–	–	–
23	9	2	–	1	–	–	–
24	10	–	–	–	–	–	–
25	11	–	–	–	–	–	–
26	12	–	1	–	–	–	–
27	13	–	–	–	–	–	–
28	14	–	–	–	–	–	–
29	15	–	–	1	–	–	–
30	16	–	–	1	1	–	–
31	17	1	1	1	1	–	–
32	18	–	2	2	1	–	–
33	19	–	1	–	–	–	–
34	20	–	–	–	–	–	–
35	21	–	–	–	–	–	–
Mortality No.		6/16	5/16	5/16	3/16	0/16	0/16
Percent %		37.5	31.2	31.2	18.7	0	0

The most prominent PM lesions appeared were tracheitis, pneumonia, air sacculitis, splenomegaly, pancreatitis, enteritis, and nephritis. Chickens of G1 were more affected than other groups in terms of frequency and severity of lesions. No thymus, bursal lesions or caseous plug at tracheal bifurcation were observed.

No clinical signs, mortalities or PM lesions were recorded in G5 and G6.

### Body weights of the experimental groups

**Fig. 1:**
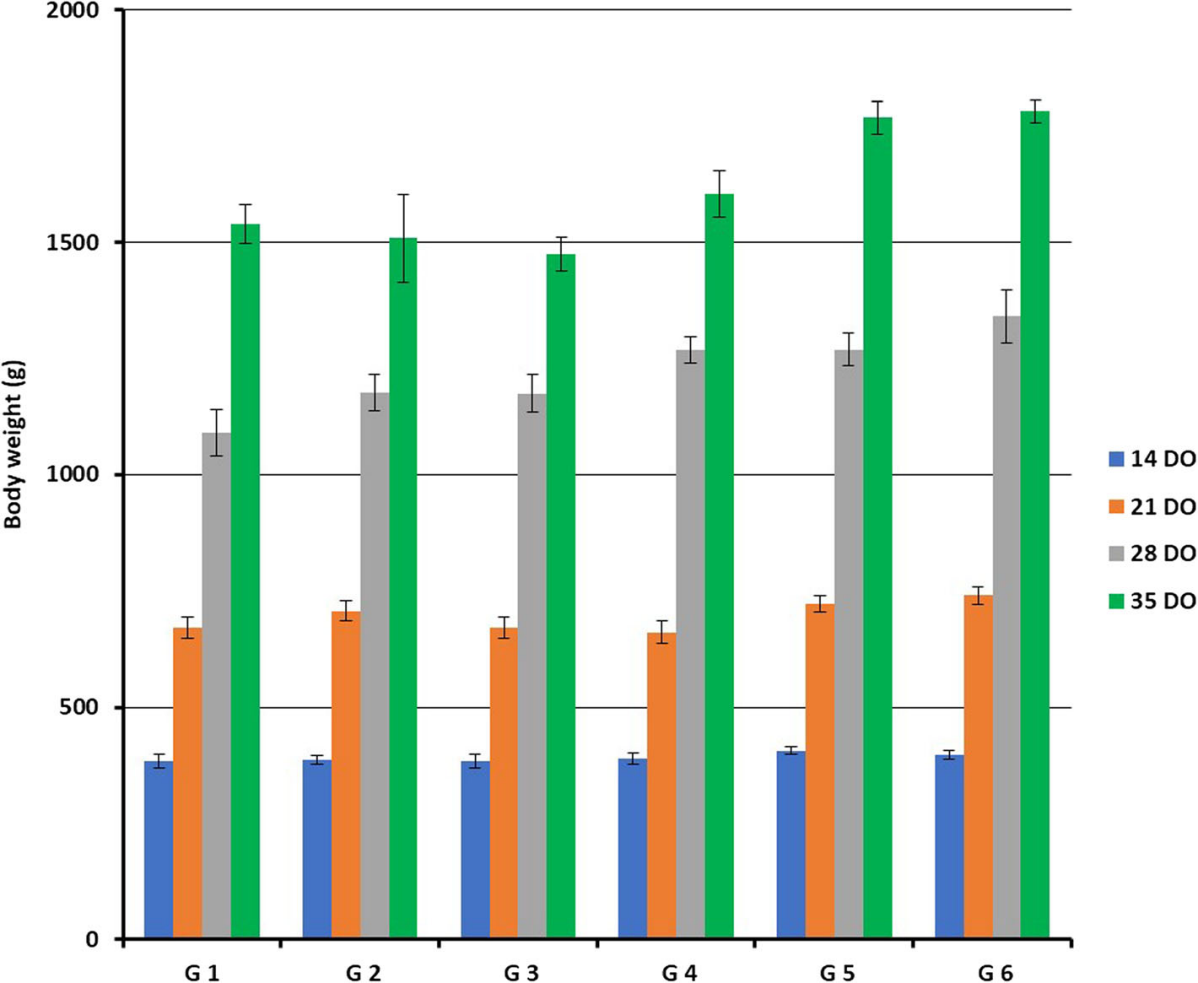
Mean ± SE body weight (gram) of all experimental chicken groups at 14, 21, 28 and 35 DO

All chicken groups had no significant difference at 14th day of age. On the other hand, a significantly decrease of body weight were observed in chickens of G1, G3 and G4 (*P* < 0.05) in compare to the birds of G2, G5 and G6 at 21th day of age. At 28th day, all vaccinated and infected chickens (G1, G2 and G3) were significantly decreased (*P* < 0.05) in body weights than G4, G5 and G6. Finally, at 35th day of age the body weights were significantly decreased (*P* < 0.05) in all H9N2 infected groups from G1 to G4 as 1540, 1509, 1474 and 1605 g, respectively than non-infected control G5 and G6 as 1769 and 1782 g, respectively (Fig. 1).

### Histopathology

**Fig. 2:**
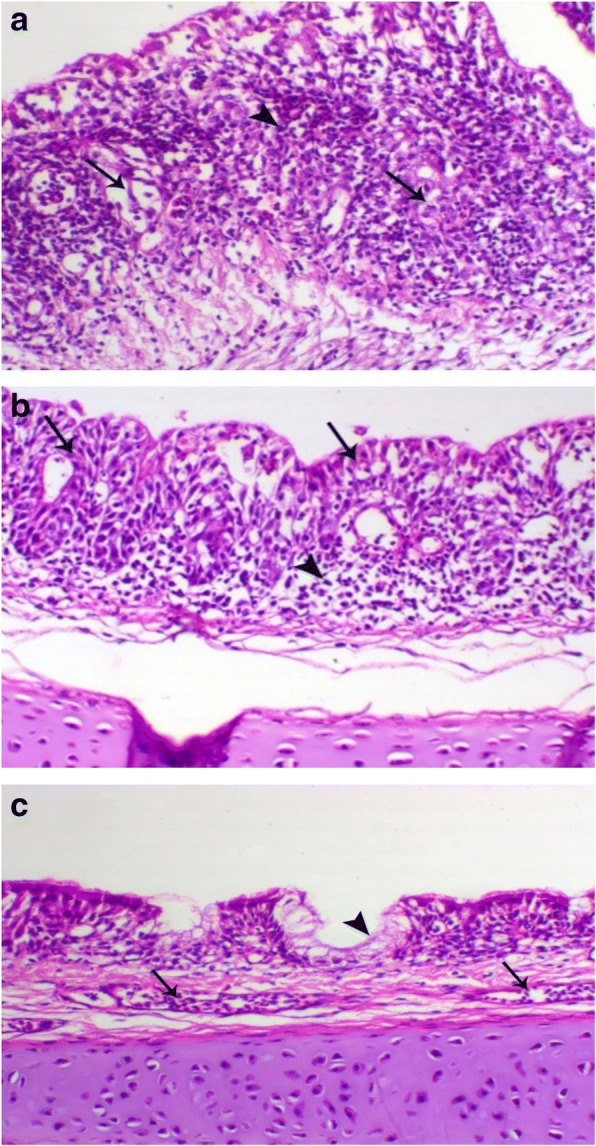
**a** Trachea of birds in G1 showed severe degeneration of the tracheal mucous glands (arrow) with marked leukocytic infiltration. H&E, X 200; **b** Trachea of in G2 showed epithelial and glandular degeneration (arrow) and lower sub-epithelial leukocytic infiltration (arrowhead), H&E, X 200; **c*** trachea* of non-infected non-vaccinated chicken G6 showed normal epithelial lining (arrowhead) contained blood vessels (arrow), H&E, X 200

Trachea of bird in G1 showed severe degeneration of mucous glands with partial loss of the lining mucosa (Fig. 2a), while other H9N2 infected groups G2, G3 and G4 showed mild to moderate epithelial degeneration and hyperplasia of the mucosal lining (Fig. 2b and c).

**Fig. 3:**
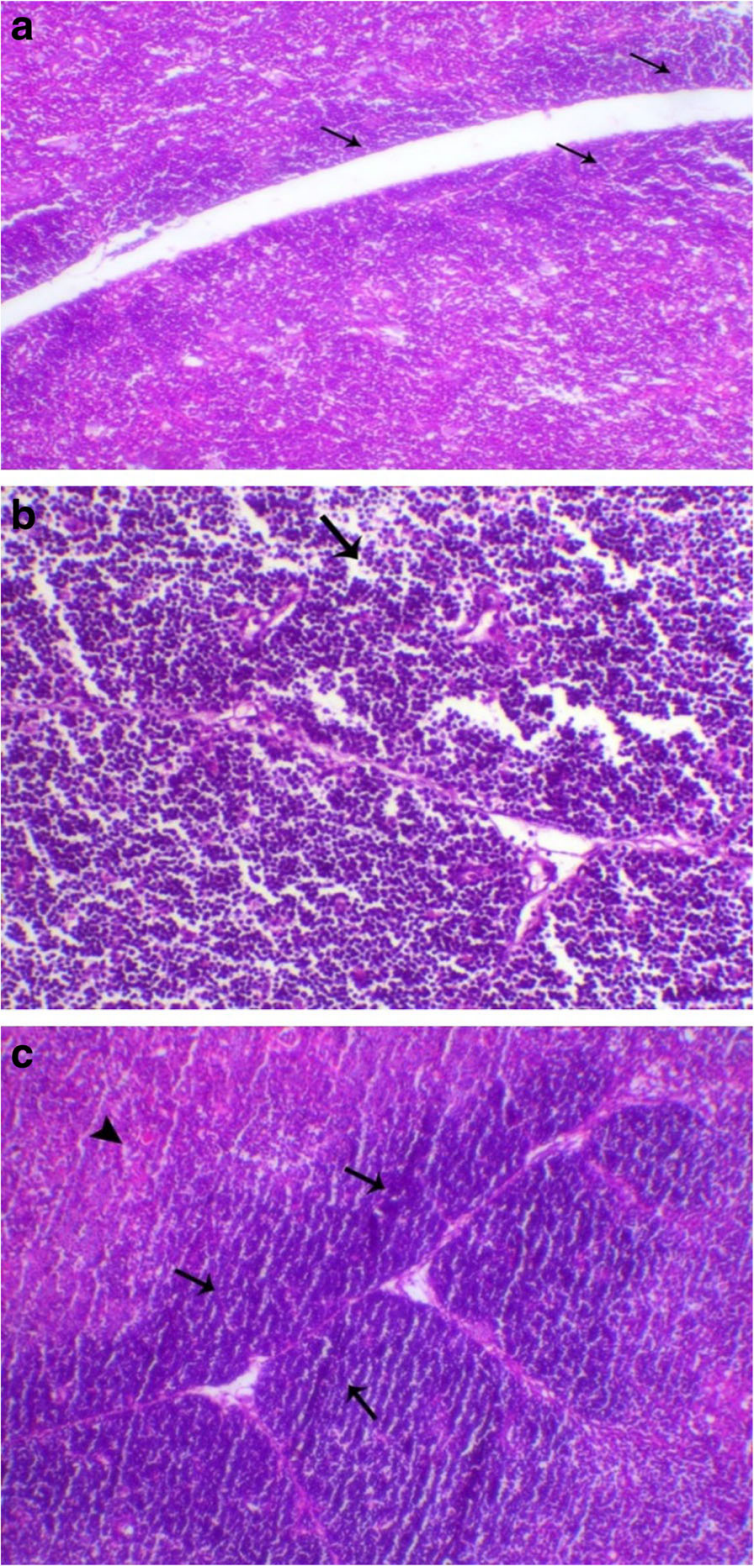
**a** Thymus of bird in G1 showed severe loss of cortical basophilic thymocytes H&E, X 100; **b** Thymus of birds in G4 showed a marked decrease of cortical thymocytes (arrow), H&E, X 200; **c** Thymus of control chicken G6 revealed normal cortex and medulla with marked distinction between each. H&E, X 200

Thymus lesions appeared as marked loss of cortical basophilic thymocytes which revealed marked lymphoid depletion was also noticed in the early infected G1 chickens (Fig. 3a). While birds of G4 showed degeneration and marked decrease of cortical thymocytes compared to control chicken G6 (Fig. 3b and c).

**Fig. 4:**
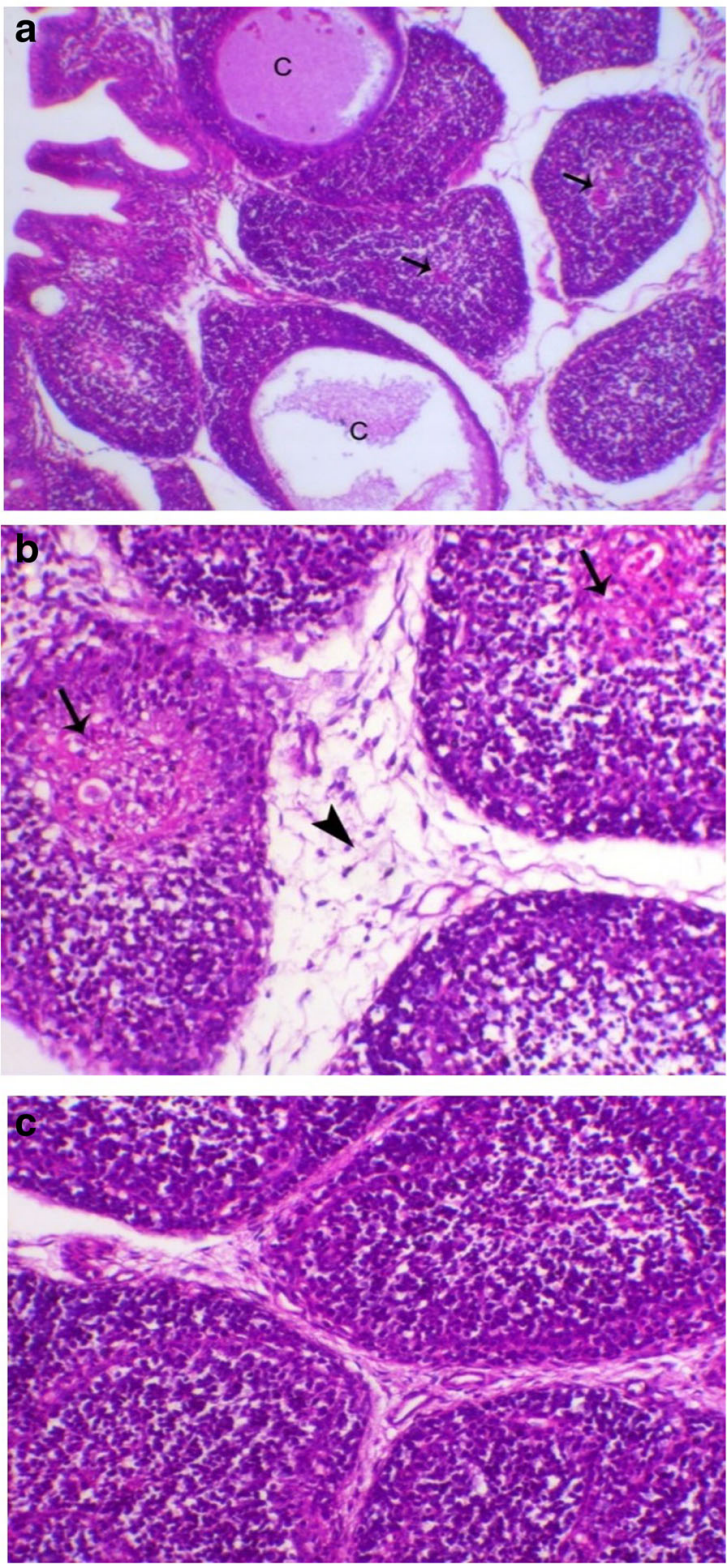
**a** Bursa of birds in G1 revealed lymphoid depletion (arrow) with large mucin filled cysts within some of follicle (**c**). H&E, X 100; **b*** bursa* of birds in G4 showed lymphoid depletion with appearance of reticular fibers in the medulla of lymphoid follicle (arrow), and with marked increase in thickness of interfollicular connective tissue (arrowhead). H&E, X 200; **c*** bursa* of Fabricius of birds in G6 showed normal bursal lymphoid follicles. H&E, X200

Bursa of Fabricius of birds of G1 (Fig. 4a) and G2 showed lesser degree of lymphoid depletion than birds of G3 and G4 (Fig. 4b) which appeared as marked lymphoid depletion with large mucin filled cysts within some follicles and thickening of the interfollicular connective tissue compared to control chicken G6 (Fig. 4c).

**Fig. 5:**
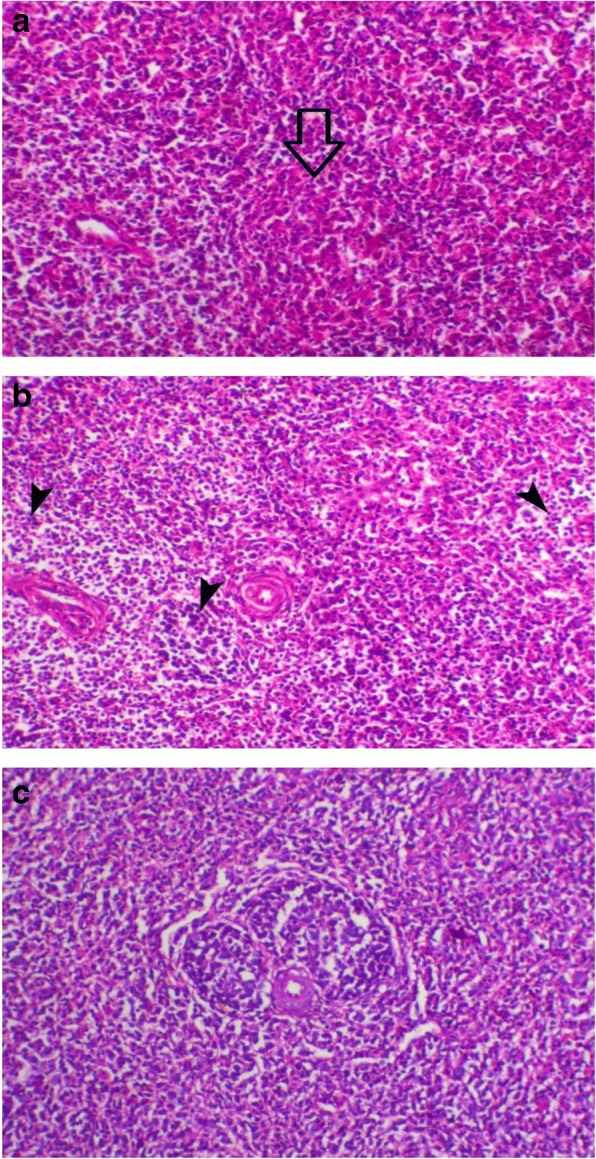
**a** Spleen of G1 birds showed absence of lymphoid follicle (arrow). H&E, X 200; **b** Spleen of birds in G4 (non-vaccinated and H9N2-infected) showed decrease of lymphoid follicle (arrowhead). H&E, X 200; **c** Spleen of birds in G6 showed normal lymphoid follicle. H&E, X 200

Spleen of chicken G1 and G4 (Fig. 5a and b) showed mild to moderate degree of lymphoid depletion, while the examined spleen of all other chicken groups was normal histology (Fig. 5c).

**Fig. 6:**
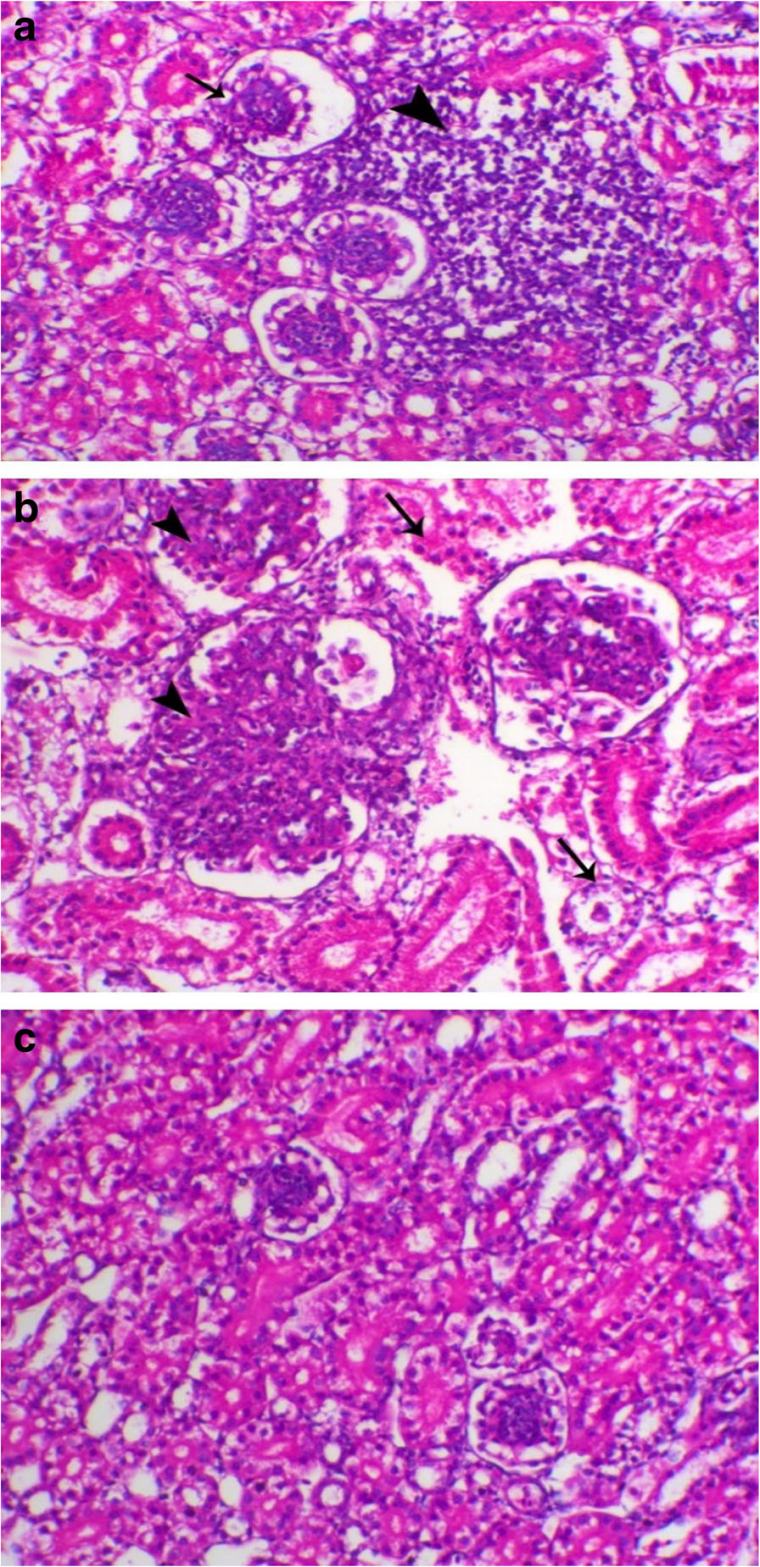
**a** Kidney of G1 birds showed periglomerular and peritubular inflammatory cells infiltrations mainly lymphocytic (arrowhead). H&E, X 200; **b** Kidney of G4 birds showed marked glomerulonephritis features which revealed loss of capillaries and hypercellularity (arrowhead), with marked degeneration of renal tubules (arrow). H&E, X 200; **c** Kidneys of normal birds showed normal renal tubular and glomerular structures. H&E, X 200

Renal inflammation of chickens G1 (Fig. 6a) and G4 showed lesions of glomerulonephritis which was obvious in most of examined birds. Interestingly, most of the lesions were glomerular rather than tubular and with a diffuse pattern with some tubular interstitial nephritis (Fig. 6b). Other chicken groups were histologically normal (Fig. 6c).

### Mean HI titers of experimental chicken groups

The mean HI titer of the 10 sacrificed birds at day 1 for H9N2 and NDV were 6.1 and 6.7 log2, respectively.

For H9N2: At 7th dpi, the HI titers of G3 were significantly (*P* < 0.05) lower than G2, G4 or G1 for (with no significant difference between samples of chicken G1 and G4). While, the only significant lower titers (*P* < 0.05) at 14th dpi were in G1 (4.7) rather than G2, G3 and G4 (6.9, 6.2 and 6.7, respectively).

Regarding NDV: At 7th and 14th dpi, the highest significant (*P* < 0.05) HI titers were G2, G3 and G5 as 4.2, 4 and 3.9 and 3.7, 3.9 and 4.2, respectively compared to the lowest significant (*P* < 0.05) titers in G4 at the same dpi as 2.8 and 0.9, respectively (Table 3).

**Table 3:** Mean ± SE of HI titers for H9N2 and NDV (Geometric Mean) in different experimental chicken groups at 7th and 14th dpi

Group	H9N2		NDV	
	7th dpi	14th dpi	7th dpi	14th dpi
1	7.1 ± 0.2b	4.7 ± 0.2b	3.8 ± 0.3ab	2.9 ± 0.3b
2	8.2 ± 0.3a	6.9 ± 0.3a	4.2 ± 0.4a	3.7 ± 0.3a
3	5.5 ± 0.4c	6.2 ± 0.5a	4.0 ± 0.3a	3.5 ± 0.3a
4	7.3 ± 0.3b	6.7 ± 0.6a	2.8 ± 0.2bc	0.9 ± 0.2c
5	2.0 ± 0.2d	0.9 ± 0.3c	3.9 ± 0.3ab	4.2 ± 0.2a
6	2.2 ± 0.2d	0.8 ± 0.2c	2.6 ± 0.4c	0.7 ± 0.2c

*n* = 10 serum samples from each group

Mean values with different letters in the same column differ significantly at *P* < 0.05

### Quantification of H9N2 virus shedding by real-time RT-PCR

**Fig. 7:**
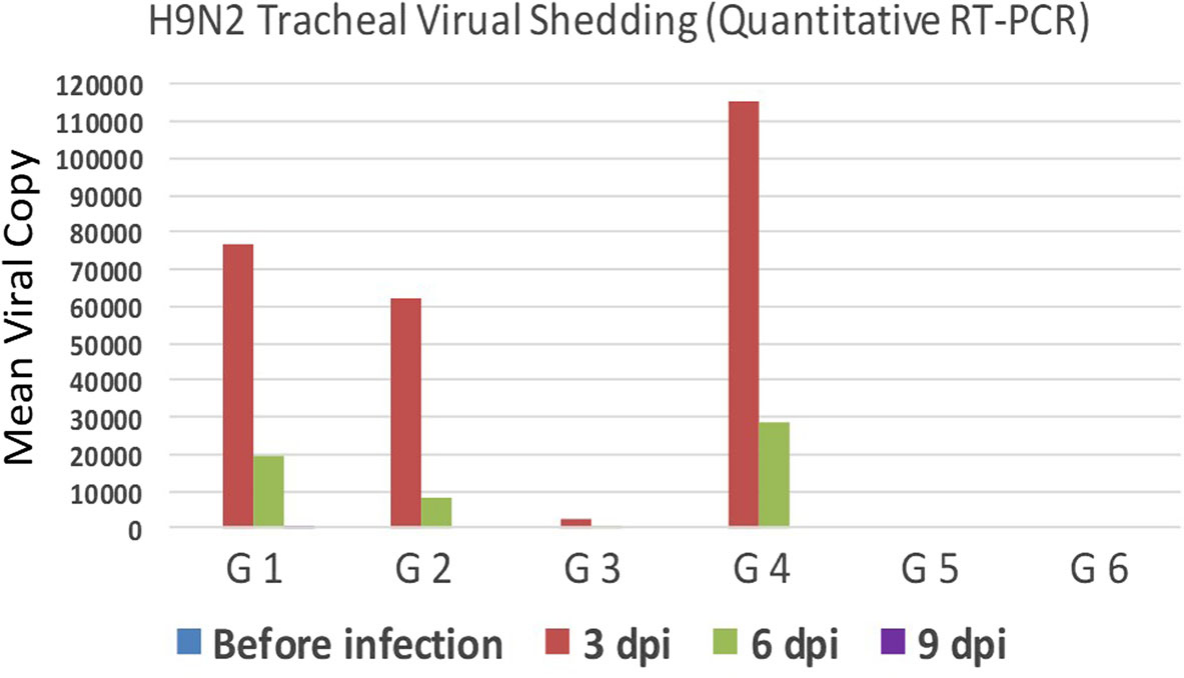
Results of H9N2 shedding (just before infection, 3, 6 and 9 dpi) using quantitative RT-PCR

Tracheal swabs collected before challenge then at 3, 6 and 9 dpi revealed that the highest viral titers were in chicken G4 followed by G1, while the least titers were in G3 at 3 dpi. Also, the shedding of the H9N2 virus was persisted for 9 dpi in G1 only (Fig. 7).

## Discussion

Co-infections between LPAI subtype H9N2 and NDV usually occur, but cannot be easily diagnosed due to confusing similar clinical signs [38]. However, H9N2 infection is mostly associated with elevated mortality and severe economic losses due to many factors such as bad management, concurrent bacterial or viral diseases, live IBV or NDV vaccines, immune suppressive agents, age at the infection and the breed of chickens, which are mostly influenced the course of H9N2 infections [1, 9, 20, 29].

In the present work, we studied the interaction between both LPAI subtype H9N2 infection and vaccination with lentogenic NDV (LaSota strain) under experimental condition.

No clinical signs were observed in G5 and G6 (non-infected groups), while infected and non-vaccinated G4 exhibited mild respiratory signs (without head swelling) and diarrhea. All groups infected and vaccinated with LaSota showed more severe clinical signs started from 3 dpi and continued until the end of the observation period except diarrhea that stopped after 7 days from its appearance. Interference between H9N2 and vNDV was studied by Bonfante, et al. [8] who reported that the H9N2 challenge made birds more susceptible to the vNDV, lowering the minimum dose required to cause an infection, exacerbating the clinical outcome, while delaying the onset of the disease and time of death.

The highest severity of signs was recorded specially in chickens of G1 at 3–6 dpi concurrently with high H9N2 viral shedding in this group compared to G4 as detected by rRT-PCR, the results are in agreement with the findings detected by Kwon, et al. [24], which observed that following infection with an LPAI H9N2 virus, the clinical sign was peaked on 6 dpi. In the present study, diarrhea and dullness of the birds were observed after 2 dpi, reached its maximum at 5 dpi and started to decline at 8 dpi, which is like the findings of Aslam, et al. [4].

Regarding the mortality, the chickens of G3 were suffered from early mortality at 2nd dpi and persisted till 11 dpi while the highest mortality (37.5%) was observed in G1. The least mortality (18.7%) was in chicken G4 began from 12th dpi and continued only for 3 days. Some studies reported no mortality after experimental infection of SPF-chickens with H9N2 [6, 8, 26].

This unusual high mortality rate in the present study may be due to the use of commercial broiler and not SPF chicks, which might be naturally carried some bacterial infection such as Mycoplasma and/or E coli, which are known to exaggerate the mortality of H9N2 infection. Li, et al. [25] found out that infection of SPF chicken with H9N2 virus did not induce any clinical signs or deaths after experimental infection, implying that co-infections with other pathogens might be the cause for these H9N2 viruses to be lethal in the field.

Also, the increase in morbidity and mortality frequency after H9N2 AIV infection could be as a result of viral dissemination and spreading from the nasal cavity, which is the primary site of infection, to the lower respiratory tract, kidneys, intestines and some visceral organs [21, 28].

A significant decrease in body weights (*P* < 0.05) at the end of the experiment was reported among all infected groups in compare to G5 and G6, where the vaccinated and infected groups (specially at the same time in G3) was more affected than that only infected with H9N2 (G4). Also, the bird weights in G4 significantly (P < 0.05) differ by 9.2 and 9.9% than G5 and G6 respectively. In contrast, Costa-Hurtado, et al. [11] reported that no significant difference in body weight between control and groups either infected with H9N2 or vaccinated with NDV (LaSota).

Post-mortem lesions were more severe in chickens of G1 than other groups. The affected organs were trachea, thymus, bursa, spleen and kidney. These finding indicated that H9N2 not only affects respiratory and digestive systems but can also affects the immune organs (thymus gland and bursa of Fabricius) and kidney especially in cases of infected and LaSota vaccinated birds. However, the respiratory organs showed more prominent gross lesions in comparison to other organs. Similar findings were observed by Taubenberger and Morens [37]; Capua and Alexander [10] Hadipour, et al. [22] as well as Subtain, et al. [35]. However, the frequent cast formation in the tracheal bifurcation, which has been reported in field cases of H9N2 avian influenza outbreaks, was not observed in this experiment.

Alexander [2] reported that more cell injuries in the tissues of the respiratory system and gastrointestinal tract were recorded after infection with LPAIV. More severe lesions in the lungs and trachea could be attributed to the presence of a trypsin-like enzyme in the respiratory mucosa.

Histopathologically, chickens of G1 had severe of tracheal epithelial degeneration and hyperplasia of the mucosal lining compared to mild to moderate degree in G2, G3 and G4 which may be related to the higher late mortality obtained in this group along with obvious renal glomerulonephritis causing renal failure. This agreed with Aslam, et al. [4] who recorded an inflamed trachea and kidney of broilers infected with LPAI H9N2 and the histopathological lesions characterized by tracheal epithelial sloughing off with severe infiltration of inflammatory cells along with severe glomerular congestion of kidney.

Also, Doustar, et al. [15] proved in an experimental study that H9N2 virus could induce apoptosis and subsequent renal tissue damage. In addition, different mechanisms by which H9N2 can cause this effect were discussed in further investigations too [34, 40].

H9N2 infected birds (G4) also showed marked lymphoid depletion in the form of degeneration of thymocytes, follicular atrophy and cystic follicles of bursa of Fabricius and splenic lymphoid depletion. Similar results were reported by Kwon, et al. [24]. In addition, EL-bayoumi, et al. [16] reported about lymphocytic depletion and decreased immune response of birds infected H5N1, H9N2, and NDV.

Interestingly, the antibody titers against NDV (LaSota- vaccine) were significantly decreased (*P* < 0.05) by the presence of H9N2 suggesting that NDV had insufficient replication to trigger the humoral response even though they were administered the typical vaccination dose of LaSota strain. Another explanation is the immunosuppressive effect of H9N2 that proved histopathologically as degeneration of thymocytes and lymphoid depletion of bursa and spleen. Also, Allawe [3] compared between LaSota vaccinated broiler chickens with or without H9N2 infection. At 40 days of age, the birds vaccinated with live NDV vaccines showed highest titers against ND in comparison with the vaccinated infected group with H9N2 that indicated the immunosuppressive effect of H9N2 infection.

Viral shedding patterns in tracheal swabs was detected for up to 9 dpi in birds of G1, while in all other groups shedding stopped on the 6 dpi. This may indicate that in case of NDV live vaccination after accidental unnoticed field infection with LPAI subtype H9N2 leads to prolonged shedding of H9N2 viruses compared with the case of NDV live vaccination before H9N2 field infection.

The least concentration of virus shedding (2630 virus copy/swab) appeared in G3 versus the highest concentration (115,300 virus copy/swab) in the non-vaccinated and infected group (G4) at 3dpi and 614 virus copy/swab compared to 28,500 virus copy/swab at 6 dpi in both groups respectively, may turn out a possibility of some mechanism of interference between NDV live vaccines and H9N2 viruses in the respiratory tract of broiler chickens.

This may be related to the fact that LaSota vaccine and LPAI viruses replicate in cells where there are trypsin-like proteases such as in the upper respiratory and intestinal epithelium, so they might compete for the same target cells or replicate in adjacent cells [31, 36]. LaSota virus binds through the Haemagglutinin-Neuraminidase (HN) glycoprotein to sialic acid-containing receptors on the cell surface, as well as the HA glycoprotein does for LPAI-H9N2 [39]. This suggests that infection with a heterologous virus may result in temporary competition for cell receptors or susceptible cells, resulting in decreased initial replication of the second virus; but as replication of the first virus declines, the second virus increases to fill the gap [11].

Also, LaSota NDV strain is known to be a weak interferon inducer as part of their low virulent phenotype profile, local interferon production might still be able to interfere with LPAIV replication [14].

The results obtained underline the importance of co-infections, which can either exacerbate clinical disease or affect virus replication by lowering viral titers to under the levels of detection and affecting serological results and in some cases prolonged the time of virus shed which could favor for further transmission and these data was supported by Costa-Hurtado, et al. [12] and Ge, et al. [19] who concluded that, co-infection with NDV and HPAI can affect the viral replication dynamics and the disease caused by these viruses in chickens, but this effect will depend on the virulence of the viruses involved, the challenge titer of the viruses and the timing of the coinfections.

## Conclusions

There was an adverse effect of combined infection with H9N2 and live LaSota vaccination in terms of clinical signs, body weights, mortality, histopathological lesions, viral shedding, and humoral immune response. Here we also recommend to diagnose the presence of H9N2 infection before ND vaccination to avoid to the exaggeration and immunosuppressive effect of H9N2 infection.

## Methods

### Experimental animals

A total of 130 one-day old commercial broiler chicks Cobb 500 purchased from a local poultry company (NASCO-Egypt) located in Alexandria governorate were used for the experimental infection in this study. All chicken groups were weighted at 14th, 21th, 28th and 35th day of age. Study design and allocating animals to experimental groups are explained in Table 4.

**Table 4:** Experimental design

Group	Vaccination with LaSota	Day of H9N2 infection	Weighting	Pooled tracheal swabs	10 serum samples/group (7th and 14th dpi)	Histopathology of 2 sacrified birds at 4th and 9th dpi
1	18 DO	15 DO	14,21,28,35 DO	15,18,21,24 DO	22,29 DO	19,24 DO
2	18 DO	18 DO		18,21,24,27 DO	25,32 DO	22,27 DO
3	18 DO	21 DO		21,24,27,30 DO	28,35 DO	25,30 DO
4	–	18 DO		18,21,24,27 DO	25,32 DO	22,27 DO
5	18 DO	–		21,28 DO	25,32 DO	22,27 DO
6	–	–		21,28 DO	25,32 DO	22,27 DO

*dpi*, days post infection, *DO* Days Old

All the experimental work, tests and procedures were complied with the general guidelines of Damanhour University and were approved by the Local Ethics Commission of the Animal Health and Welfare of Damanhour University with respect to care of animals under study and all efforts were made to minimize suffering. The Ethical Approval Code was DMU/VetMed-2017/0023.

### Housing and husbandry

Chicks were housed in galvanized wire cages (40 cm high × 50 cm width × 100 cm length) in (ten chicks each). Fresh feed and water were offered ad-libitum through the experimental period. All chicks were kept under the same managerial, hygienic and environmental conditions. Chicks were fed to cover their requirements according to NRC [38]. The sacrificed birds all over the experiment were previously anesthetized using sodium pentobarbital (50 mg/kg) by intraperitoneal injection to minimize suffering.

### Experimental procedures

Vaccines. Birds of G1–4 were vaccinated against NDV by eye drop between days 15th to 21st (Table 4) using LaSota® (Schering plough, USA).

#### Challenge virus

Egyptian H9N2 strain isolated from broiler chickens in Alexandria Governorate (A/Chicken/Egypt/93/2015 and the GenBank accession No: KY872759) was used as a challenge virus in a dose 0.2 ml/bird of 10^6.3^ EID50/dose (0.1 ml by nasal instillation and 0.1 ml through eye drop) at 18th day of age [30].

### Sample size

Ten chicks were sacrificed at the first day of age and serum samples were used to determine the level of maternally derived antibodies against H9N2 and NDV using Haemagglutination inhibition test. The remaining 120 chicks were distributed randomly into 6 equal groups of each 20 chicks. Birds were weighed and sacrificed by exsanguination after prior anesthesia.

### Experimental outcomes

All the birds were examined daily for the following parameters:

- ***Clinical signs, Mortality rates and Post-mortem (PM) lesions***. Birds in all groups were examined daily up to 35 days of age and all disease signs, mortalities and PM lesions of dead or sacrificed birds were recorded.
- ***Histopathological examination***. Two birds from each group were sacrificed under anesthesia with intravenous injection of sodium pentobarbital (50 mg/kg) at 4th and 9th dpi in groups (1–4) and at 22 and 27 days of age in groups 5 and 6. Samples from trachea, thymus, bursa, spleen and kidney were collected, fixed in 10% formalin solution, stained with Hematoxylin and Eosin (HE) and examined for lesions [5].
- ***Haemagglutination inhibition (HI) test***. Serum samples collected at 7th and 14th dpi were tested by HI test for H9N2 and ND antibodies using standard haemagglutination antigens (2^8^ titers) for both viruses. HI test was applied using 4 Haemagglutination Units (HAU) of the relevant antigens and 25 μl of 1% chicken RBCs according to Alexander [2] and the results were analyzed using SPSS
- ***Quantification of H9N2 virus shedding by Real-Time RT-PCR (rRT-PCR).*** Pooled tracheal swabs were collected from 10 chickens in each group just before H9N2 infection then at day 3, 6 and 9 post H9N2 infections. The RNA was extracted using QIAamp viral RNA Mini-kits (QIAGEN 52904- Germany) and the test was performed using rRT-PCR (Strategen 3005P USA) with Quantitect RT-PCR Kit reaction buffer (QIAGEN 204443- Germany) and positive control RNA from A/quail/Egypt/113413v/2011(H9N2). The Sequence of H9 primers and probe as published by Ben Shabat, et al. [7] were used.
- Thermal profile was RT at 45 °C for 10 min and 95 °C for 10 min, followed by 40 cycles of PCR at 95 °C for 15 s for denaturation and 60 °C for 45 s for annealing and extension.

### Statistical methods

Data of birds’ body weight were subjected to analysis of variance procedures appropriate for a completely randomized design using the GLM procedures of SPSS (2008). The differences among means were determined using the student Newman keuls test. Statements of statistical significance are based on *p* < 0.05.

## Abbreviations

AIV: Avian influenza viruses
dpi: Day post infection
dpv: Day post vaccination
IBV: Infectious bronchitis virus
NDV: Newcastle disease virus
PM: Post-mortem
SPF: Specific pathogen-free
